# Predicting working memory efficiency across adulthood: the role of enhancement and suppression attentional mechanisms, moderated by age and other factors

**DOI:** 10.1007/s40520-025-03059-8

**Published:** 2025-05-28

**Authors:** Elissa López-González, Ulises Caballero-Sánchez, Ixchel Gómez-González, Mónica Méndez-Díaz, Oscar E. Prospéro-García, Alejandra E. Ruiz-Contreras

**Affiliations:** 1 Present Address: Laboratorio de Neurogenómica Cognitiva, Unidad de Investigación de Psicobiología y Neurociencias, Coordinación de Psicobiología y Neurociencias, Facultad de Psicología, Universidad Nacional Autónoma de México (UNAM), Mexico City, Mexico; 2 Laboratorio de Cannabinoides, Departamento de Fisiología, Facultad de Medicina, UNAM, Mexico City, Mexico

**Keywords:** Cognition, Working memory, Attention, Mental health, Cognitive reserve, Sleep

## Abstract

The global population of individuals aged 60 years and older now exceeds one billion and continues to grow, underscoring the necessity of addressing cognitive challenges associated with aging. The present study aimed to evaluate the enhancement and suppression of attentional mechanisms, as well as working memory efficiency (WME), across the adult lifespan. Additionally, it examined factors that may moderate these relationships. A total of 194 participants, aged between 20 and 80 years, completed questionnaires and participated in an experimental task designed to assess attentional mechanisms and WME. The results indicated that both enhancement and suppression mechanisms remained stable throughout adulthood; however, WME showed a decline with increasing age. Notwithstanding, several moderating variables influenced these outcomes. Cognitive reserve and current cognitive functioning were found to be positive predictors of WME, whereas depression and anxiety exerted negative effects. Furthermore, a low level of cognitive reserve uniquely predicted diminished suppression of irrelevant information with advancing age. WME was consistently lower across all ages among participants with high levels of depression, in contrast to those with low or moderate levels, in whom WME followed the expected age-related trajectory. Additionally, elevated anxiety levels and an average sleep duration of five hours were associated with reduced WME, particularly in relation to the enhancement mechanism. Conversely, low levels of depression were linked to improved WME via the enhancement mechanism. These findings underscore the importance of moderating factors that may mitigate cognitive decline or support cognitive functioning throughout the aging process.

**Supplementary Information:**

The online version contains supplementary material available at 10.1007/s40520-025-03059-8.

## Introduction

The global population of individuals aged 60 years and older has surpassed one billion, representing 9.9% of the world’s total population. With the continuous increase in life expectancy, this demographic is projected to constitute 16.4% of the global population by the year 2050 [1]. This demographic transition poses significant challenges to the health and quality of life of older adults. Healthy aging is defined as the process of fostering and preserving functional ability to ensure well-being in later life [1]. A common condition associated with aging is the onset of mild cognitive impairment or dementia, including Alzheimer’s disease. Nonetheless, there remains a strong aspiration among individuals to reach old age while minimizing or preventing cognitive decline and managing brain pathology [2].

Researchers have concentrated on identifying factors that promote healthy aging and safeguard against cognitive decline, including anxiety, depression [3, 4], sleep deprivation [5], and limited educational attainment [6]. Conversely, engagement in complex work, as well as physical, leisure, and cognitive activities throughout life, has been associated with the maintenance of cognitive health [2]. Cognitive domains that are frequently impacted by cognitive impairment include working memory (WM), attention, executive functions, and processing speed [3]. Nevertheless, there remains a lack of comprehensive understanding regarding the progression of these processes throughout adulthood.

Visual scenes present multiple stimuli that must be processed by the inherently limited human cognitive system. Consequently, attention enables the concentration on regions of the scene containing relevant information [7, 9, 10], while disregarding distracting elements [11, 12]. To account for the effective selection and processing of visual information, the mechanisms of attention have been described in terms of enhancement and suppression [13, 14]. The enhancement mechanism involves a neural network that demonstrates increased activity when processing relevant stimuli, as opposed to a passive observational state. In contrast, the suppression mechanism is characterized by a reduction in neural activity below that of a passive state in regions responsible for processing irrelevant stimuli, indicating an active inhibition of such information. Both mechanisms function to prioritize stimuli pertinent to the task at hand [13, 14]. When the suppression mechanism is ineffective, distraction ensues, resulting in a shift of focus toward irrelevant stimuli instead of, or beyond, the relevant ones, thereby impairing working memory efficiency (WME) [14–17]. Eye movements constitute an objective psychophysiological indicator of both the selection of relevant stimuli and the attentional capture provoked by distractors [8–10, 18, 19].

WM enables the maintenance and manipulation of information, even in its absence, and facilitates its updating in support of goal-directed behavior [20, 21]. WME declines with age [15–17, 22–25]. One hypothesis suggests that attentional failures are predictive of WME [15–17, 23, 25]. A plausible explanation for this decline is the diminished effectiveness of the suppression mechanism in older adults compared to younger individuals [16, 17, 23, 26]. Numerous studies have demonstrated that attentional capacity decreases with advancing age [15, 17, 23, 26, 27]; however, there is limited empirical evidence examining the enhancement and suppression mechanisms across young, middle-aged, and older populations.

This study pursued three primary objectives. First, to determine whether age predicts the enhancement and suppression mechanisms of attention and WME [1]. Second, to examine whether attentional mechanisms predict WME [2]. Finally, to investigate which factors moderate age-related changes in predicting the effect of attentional mechanisms on WME [3]. It is anticipated that higher scores on factors such as cognitive reserve, years of education, and current cognitive status will predict better suppression of irrelevant stimuli and greater WME [4]. Conversely, higher levels of depression and anxiety are expected to be associated with reduced suppression of irrelevant stimuli and lower WME [5]. Furthermore, it is predicted that, in addition to age, other variables will moderate the relationship between attentional mechanisms and WME [6].

## Method

### Participants

Two-hundred and thirty-six adults aged between 20 and 85 years participated in the study after providing informed consent. The sample size was determined for a linear multiple regression analysis, assuming a medium effect size (0.15), α = 0.001 (to minimize the size of Type I error), a power of 0.9, and three predictors (age, attentional mechanism, and a moderator factor). This yielded a required minimum sample size of 182 individuals. Forty-two participants were eliminated from the analyses for any of the following causes: 10% or more non-responses during the experimental task, the lack of quality of the eye-tracking, or an incomplete response in any questionnaire. The final sample consisted of 194 (107 were female), a larger sample than those expected by the sample size calculation.

The inclusion criteria required participants to be right-handed (as assessed by the Edinburgh Inventory [28]), to have completed at least seven years of formal education (the average in Mexico [29]), and to have no history of neurological or psychiatric illness, nor any first-degree relatives with such conditions. Participants who reported chronic illnesses also indicated that these were controlled with medication. All participants reported living independently. Finally, only participants with reliable internet signals were included in the study (https://www.speedtest.net/es), because the experimental session was run online.

The study was approved by the Research Ethics Committee in accordance with the Declaration of Helsinki and the Official Mexican Standard NOM-012-SSA3-2012, which outlines ethical criteria for human research. Data collected from participants were anonymized and treated as confidential. No identifying information or images are included in this manuscript.

### Instruments

Two types of instruments were applied: to meet the inclusion criteria and for data acquisition. To assess the inclusion criteria, the following inventories were administered: the Edinburgh Handedness Inventory [28], a general demographic questionnaire, the DSM-V Substance Use Disorder Questionnaire [30], and the Montreal Cognitive Assessment (MoCA) [31]. For data collection, the following instruments were used: the Cognitive Reserve Index Questionnaire (CRIq) [32]; the State-Trait Anxiety Inventory (STAI) [33] for anxiety symptoms; and the State-Trait Depression Inventory (ST-DEP) [34] for depressive symptoms. In addition, participants reported their total years of formal education, typical sleep duration, and the number of hours they slept the night prior to the experimental session.

### Materials and equipment

The experimental sessions were conducted using the Zoom video conferencing application (Zoom Video Communications Inc., 2021), accessed from each participant’s personal computer equipped with internet access, a numeric keypad, and a webcam. Participants completed the questionnaires using either a computer mouse or touchpad, and responded to the experimental task using the numeric keypad. The experimental task required a minimum internet bandwidth of 10 Mbps. The task was presented using the RealEye online platform, which also facilitated the collection of behavioral data—including accuracy (percentage of correct responses), reaction times, and eye movement data. Specifically, RealEye uses webcam-based eye-tracking technology that relies on artificial intelligence to detect the participant’s face and pupils and predict the point of gaze on the screen [35].

RealEye includes a quality check for each participant, assessing the accuracy and validity of the eye-tracking data (e.g., data availability across trials, optimal sampling rate, data integrity, and number of gaze points outside the screen). In this regard, only participants whose RealEye qualification was good, very good or excellent were included in the data analysis. Prior studies have shown that RealEye can predict gaze fixation with an accuracy of approximately 110 pixels and a sampling frequency of up to 60 Hz [36], supporting its reliability for tracking eye movements.

### Stimuli

Grayscale images of faces (60) and scenes (60) were presented against a dark gray background (RGB: 170, 170, 170). The facial stimuli consisted of 30 female and 30 male faces obtained from the validated Karolinska Directed Emotional Faces (KDEF) database [37]. Of these, 20 faces (with an equal gender distribution) displayed positive emotional valence, 20 negative, and 20 neutral expressions. Likewise, 60 images of indoor and outdoor scenes (30 each), all exhibiting neutral valence and previously validated [38], were presented as scenes stimuli.

### Experimental task

**Fig. 1:**
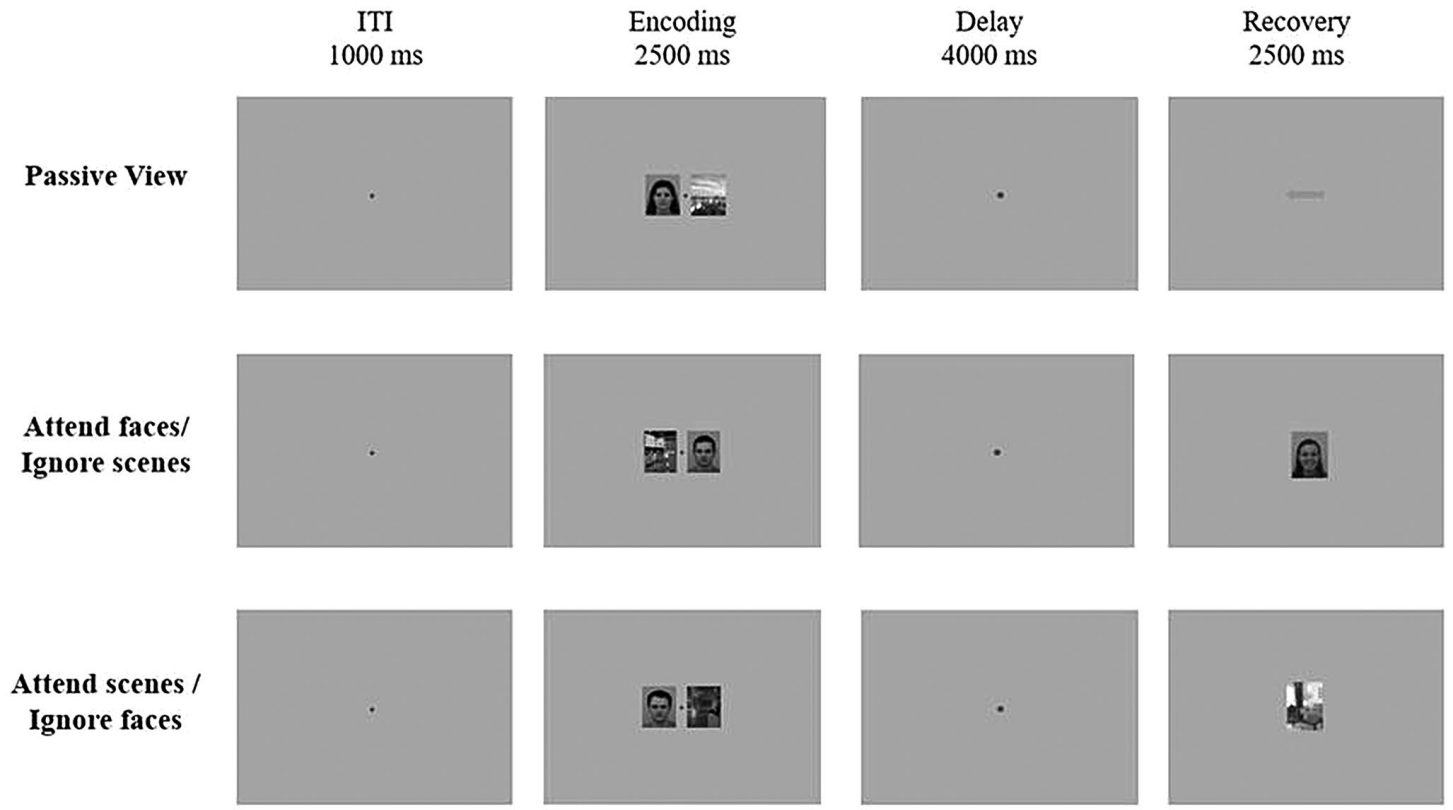
The experimental task comprised three conditions, each involving the simultaneous presentation of a face and a scene during the encoding phase. In the passive view condition, participants responded to the direction of an arrow during the retrieval phase, without selecting or memorizing any stimulus. In the attended conditions, participants were instructed to focus on one stimulus type (e.g., faces), ignoring the other (e.g., scenes), and to indicate whether the target stimulus in the retrieval phase matched that shown during encoding. Each condition consisted of 60 trials

The experimental task consisted of three conditions, divided into three independent blocks of 60 trials. The order of three conditions were equally balanced across participants. Figure 1 depicts the sequence of one trial in each of the three experimental conditions. The conditions were: (1) Passive view; (2) Attend faces/ignore scenes; (3) Attend scenes/ignore faces. In each trial, there were three phases for each condition: encoding, retention, and retrieval.

In the attend/ignore faces conditions, participants focused on one type of stimulus while disregarding the other. In contrast, for the passive view condition, participants were instructed to simply observe the images without attempting to retain them. Each trial began with the presentation of a central fixation cross (“+”) for 1000 ms. This was followed by the simultaneous presentation of a face and a scene at the center of the screen, where one was designated as the target and the other as the distractor, depending on the condition. This display lasted 2500 ms.

A retention interval followed, during which a fixation circle was displayed at the center of the screen for 3500 ms. In the retrieval phase, the target stimulus from the encoding phase was presented again, depending on the condition. Participants were required to indicate whether the retrieved stimulus matched the one previously shown: pressing the left arrow key if it was the same, or the right arrow key if it was different. For each attend/ignore faces condition, 50% of the trials included new stimuli and 50% repeated those from the encoding phase.

In the passive view condition, participants were presented with a horizontal arrow during the retrieval phase and were asked to indicate its direction: pressing the left arrow key for a left-pointing arrow, and the right arrow key for a right-pointing arrow. The direction was balanced across trials (50% left, 50% right). Participants had 2500 ms to respond.

The sequence of trials was identical for all participants. Key stimulus features were counterbalanced across blocks, including sex (female vs. male), facial valence (positive vs. neutral vs. negative), scene type (indoor vs. outdoor), arrow direction (left vs. right), and trial type (target vs. non-target). Each face stimulus, defined by sex and valence, was presented once as a target and once as a non-target in each condition. The trial sequence was semi-randomized, with the following constraints: no more than three consecutive trials could share the same facial sex, valence, or target status.

### Eye-tracking measurement

At the beginning of the task, the RealEye system calibrated the participant’s webcam using a point-based eye-tracking procedure to determine gaze direction on the screen. As facial images were the primary stimuli to be attended, ignored, or passively viewed, areas of interest (AOI) were defined around the faces, extending 5% beyond each edge of the image (left, right, top, and bottom) for improved precision. Gaze duration on the AOIs was measured for each condition. Additionally, enhancement and suppression indexes were computed (see Sect. 2.7.1. *Attentional Mechanisms*).

### Procedure

The study was conducted over two sessions. In the first session, participants provided informed consent and completed the screening instruments used for the inclusion criteria. The second session involved the execution of the experimental task (including eye-tracking recording) and the administration of the data acquisition inventories (see Sect. 2.2. *Instruments*).

### Statistical analyses

Descriptive statistics were reported using means and standard deviations for continuous variables such as age and years of schooling. For instruments such as CRIq, MoCA, STAI, and ST-DEP, medians, minimums, and maximums were reported, due to the nature of their respective measurement scales.

To analyze the data, general linear models were employed. Variables from the experimental task and the psychological inventories were standardized using z-scores, calculated based on the data from all participants.

#### Attentional mechanisms

For each condition, enhancement and suppression mechanisms were calculated using the total gaze duration on the faces, as defined by the areas of interest (AOI). The enhancement index was computed as the gaze duration on faces in the *attend faces* condition minus the gaze duration in the *passive view* condition. A positive enhancement index indicates a stronger attentional engagement with the target stimulus. Conversely, the suppression index was calculated as the gaze duration on faces in the *passive view* condition minus the duration in the *ignored faces* condition. A positive suppression index reflects a greater degree of attentional suppression for non-target stimuli.

#### Working memory efficiency (WME)

WME was measured using the inverse efficiency index (IEI), calculated for faces and scenes; it is a relative score that combines the accuracy [*d’*; for discriminating the old stimulus presented during the encoding phase (i.e., targets), against the new ones (non-targets)] and the mean reaction times (RT) for hits (i.e., targets correctly identified as targets) and false alarms (i.e., non-target incorrectly detected as target). Thus, increasing RT or decreasing accuracy would generate higher IEI [39]. The IEI is calculated by dividing the mean RT by *d’* [39]. Briefly, *d’* is a discrimination index calculated using the formula: *d ’* = ZHit–ZFalse Alarms. According to the signal detection theory, there are four possible outcomes: a hit is the target (old stimulus) correctly detected as old; a miss is a target detected as new stimulus; a false alarm is the non-target (new stimulus) detected as old; and a correct rejection is a non-target detected as new. The proportion of hits (hits/hits + misses), and false alarms [false alarms/(false alarms + correct rejections) were calculated, followed by the identification of the Z values. The Z transformation was performed with the statistical formula NORMSINV(Hit)–NORMSINV(FA) in a Microsoft Excel spreadsheet. Perfect scores (all targets correctly detected) were adjusted using these formulas: 1 − 1/(2n) for hits, and 1/(2n) for zero false alarms [40]. *Thus*,* a larger IEI means a lower WME.* Here forward, WME was described according to the IEI. It was obtained for both conditions: attend vs. ignore faces.

#### Age predicting attentional mechanisms and WME

To evaluate the predictive role of age on attentional mechanisms and WME, simple linear regression analyses were conducted. Age served as the predictor variable for the enhancement index, suppression index, and WME.

#### Attentional mechanisms predicting WME

Separate simple linear regressions were performed to assess whether attentional mechanisms predicted WME. Specifically, the enhancement mechanism was used to predict WME in the *attend faces* condition. The suppression mechanism was used to predict WME in the *ignore faces* condition.

#### Moderating variables on the attentional mechanisms, WME, and its relationship

Moderation analyses were run to assess the effect of age and other factors contributing to attentional mechanisms, WME, and their relationship. The moderators considered here were years of schooling, CRIq, MoCA, STAI, ST-DEP, usual sleep hours, and sleep hours the night before the session. When a significant interaction between the predictor and the moderator variables was detected, or when a moderator had an effect, the sample was segregated among three groups to be compared: the low (-1 standard deviation (SD)) medium (between − 1 SD to + 1 SD) and high (+ 1 SD) groups, as others have done [41]. The false discovery rate for multiple comparisons was corrected with a threshold of *p* < 0.045 according to [42]. Reported probabilities from these analyses are the Benjamini-Hochberg adjusted p-value. All statistical analyses were performed with RStudio.

## Results

### Demographic data

The descriptive data of the entire sample is presented in Table 1, as well as the mean and standard deviation of the enhancement and suppression indices and WME (IEI).

**Table 1:** Mean and standard deviation or median and minimum and maximum values, in parentheses, of the descriptive data for Raw scores of the whole sample; and the enhancement and suppression indices and WME (IEI) are presented for attend faces and ignore faces conditions

Descriptive variables	Score
Age (mean ± SD)	43.57 ± 14.75
Years of schooling (mean ± SD)	19.18 ± 4.01
Cognitive Reserve (median (min-max))	114 (86–166)
Cognitive Function (median (min-max))	27.5 (24–30)
Trait Anxiety (median (min-max))	37 (20–68)
State Anxiety (median (min-max))	33 (20–63)
Trait Depression (median (min-max))	34.5 (19–69)
State Depression (median (min-max))	41 (24–67)
Usual sleep hours (mean ± SD)	6.77 ± 1
Sleep before session (mean ± SD)	6.84 ± 1.08
**Attentional mechanisms and WM variables**	**Score**
Enhancement Index (mean ± SD)	86.55 ± 295.08
Suppression Index (mean ± SD)	52.29 ± 242.22
Working Memory Efficiency in Attend Faces (IEI; mean ± SD)	402.66 ± 153.12
Working Memory Efficiency in Ignore Faces (IEI; mean ± SD)	440.9 ± 157.32

### Do the attentional mechanisms predict working memory efficiency?

The enhancement or suppression mechanisms did not predict WME (*p* = 0.252 and *p* = 0.96, respectively; Supplementary Fig. 1).

### Does age predict the attentional mechanisms and WME?

Age did not predict enhancement or suppression indices (*p* > 0.05). However, Age directly predicted IEI (i.e., larger IEI means lower WME) for both to attend (R^2^ = 0.16, β = 0.41, *p* = 4.35 × 10^− 8^, power = 0.79) and to ignore (R^2^ = 0.14, β = 0.39, *p* = 1.88 × 10^− 7^, power = 0.59) faces conditions; thus, having more years of age predicted lower WME (Supplementary Fig. 2).

### What factors moderate the relationship between age and the attentional mechanisms, and age and the WME?

Supplementary Table 1 presents the moderation analysis results using age as the predictor variable, and attentional mechanisms or WME as the predicted ones; these analyses included years of schooling (YS), current cognitive function (CogFun), cognitive reserve (CogRes), trait (TAnx) and state (SAnx) anxiety, trait (TDep) and state (SDep) depression, usual sleep hours (S/usual), and sleep hours the night before the session (S/session) as moderators. Here, only significant moderators for attentional mechanisms or WME are reported.

#### Moderators for the age and the enhancement mechanism relationship

Age or any other moderator did not predict the enhancement mechanism (Supplementary Table 1).

#### Moderators for the age and the suppression mechanism relationship

**Fig. 2:**
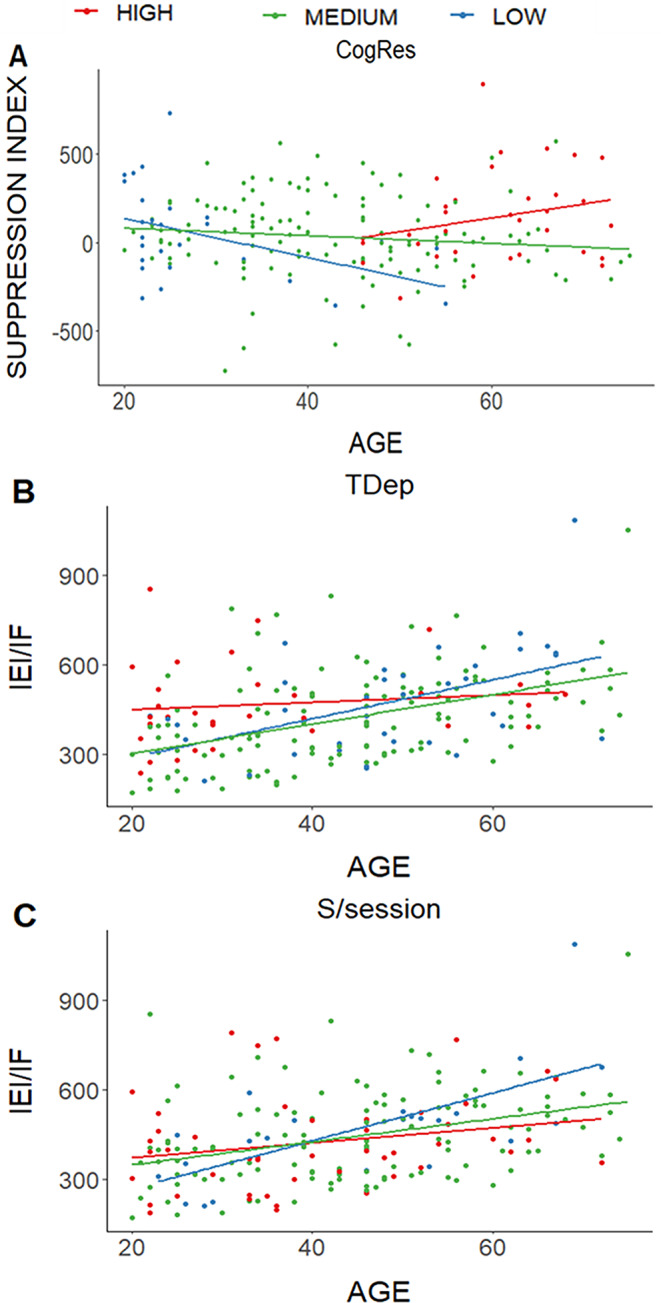
Moderation results of the significant interactions. (**A**) Cognitive reserve (CogRes) and Age interacted to predict the suppression index; only the low CogRes group showed that the suppression index decreased as age increased. (**B**) Age predicted the IEI for the ignore faces condition, moderated by trait depression (TDep); only for low and medium TDep groups, the simple linear regressions were significant; as age increased, IEI increased. In the high TDep group, no changes in IEI were observed as a function of age. (**C**) Age predicted the IEI for the ignore faces condition, moderated by sleep hours the night before the session (S/session); only for low and medium S/session groups, the simple linear regressions were significant; as age increased, IEI increased. Graphs were performed with z-scores. For better understanding, X- and Y axes showed the raw data, corresponding ± 1SD of z-scores on X-axis, and ± 2SD of z-scores on Y-axis

Moderation analysis showed an interaction between age and cognitive reserve (CogRes; β = 0.23, *p* = 0.014; R^2^ = 0.06, *p* = 0.01, power = 0.76; Fig. 2A; Supplementary Table 1). Thus, simple linear regressions were calculated to test if the slopes from low, medium, and high CogRes groups differed from zero as a function of age. Only for the low CogRes group (Mean ± SD, 92.78 ± 2.5), age significantly and negatively predicted the suppression mechanism (β= -0.28, *p* = 0.023). No other moderators affected the suppression mechanism.

#### Moderators for the age and the WME relationship

CogRes negatively predicted the IEI during the attend faces condition (β= -0.23, *p* = 0.018; Supplementary Table 1; Supplementary Fig. 3A), as the CogRes increased, IEI decreased. No interaction with age was observed (*p* = 0.672).

CogFun negatively predicted the IEI during the attend faces condition (β= -0.19, *p* = 0.02; Supplementary Table 1; Supplementary Fig. 3B), showing that as current CogFun increased, IEI decreased. CogFun did not interact with age (*p* = 0.659).

SAnx positively predicted the IEI during the attend faces condition (β = 0.132, *p* = 0.048; Supplementary Table 1; Supplementary Fig. 3C), observing that as SAnx increased, IEI increased. In addition, SAnx positively predicted the IEI during the ignore faces condition (β = 0.139, *p* = 0.04; Supplementary Table 1; Supplementary Fig. 3D), observing that as SAnx increased, IEI increased. SAnx did not interact with age (Attend faces: *p* = 0.59 and Ignore faces: *p* = 0.74, respectively).

TDep significantly interacted with age (β= -0.17, *p* = 0.04; Fig. 2B; Table 2; Supplementary Table 1) for predicting IEI in to the ignore faces condition, which explains a portion of the variance (R^2^ = 0.19, *p* = 3.27 × 10^− 7^, power = 0.86). Only for low and medium TDep groups, the simple linear regressions were significant (low: 23.21 ± 1.81, β = 0.59, *p* = 0.004; and medium: 35.7 ± 6.1, β = 0.46, *p* = 1.1 × 10^− 7^; high: *p* = 0.69); as age increased, larger IEI. Further analysis showed that no differences were found in the IEI of ignore faces condition between younger (20–35 years) against the older participants (> 50 years; *p* = 0.435) in the high TDep group; likewise, younger participants of the high TDep group had higher IEI for to ignore faces condition than younger participants from the low and medium groups (*p* = 0.0013). The same pattern of results was found for the IEI for the attend faces condition (younger and older participants of the high TDep group did not differ in their IEI, *p* = 0.296; but younger participants of the high TDep group had lower IEI than the low and medium TDep groups, *p* = 0.007; however, the interaction between TDep and age for IEI for the attend faces condition were not significant).

**Table 2:** Results of the moderation analysis with both enhancement and suppression attentional mechanisms as the predictor variables for IEI, the predicted variable. The moderators analyzed, independently, were years of schooling, current cognitive function, cognitive reserve, trait and state anxiety, trait and state depression, usual sleep hours, and sleep hours the night before the session. Results are presented alongside moderating factors. The R^2^ and p-value of the statistically significant models are indicated in bold, the significant effects of age and the significant interactions or the effect of the moderator alone are also indicated in italics

Moderation	Enhancement - IEI/AF		Suppression - IEI/IF	
	Β	*p*	β	*p*
**Years of schooling**				
AttMech	-0.105	0.287	-0.013	0.952
YS	-0.075	0.547	-0.056	0.672
Age	*0.408*	*1.41E-07*	*0.371*	*2.55E-06*
AttMech: YS	-0.015	0.948	0.044	0.731
AttMech: Age	0.014	0.952	0.064	0.624
YS: Age	-0.029	0.870	0.012	0.952
AttMech: YS: Age	-0.027	0.848	0.062	0.611
MODEL (R²/p)	***0.161***	***5.24E-06***	***0.137***	***5.30E-05***
**Cognitive Reserve**				
AttMech	-0.104	0.424	-0.081	0.571
CogRes	*-0.222*	*0.026*	0.009	0.968
Age	*0.557*	*6.48E-08*	*0.345*	*0.002*
AttMech: CogRes	0.050	0.800	0.052	0.773
AttMech: Age	-0.031	0.904	-0.014	0.952
CogRes: Age	0.043	0.755	0.048	0.095
AttMech: CogRes: Age	-0.013	0.952	0.096	0.379
MODEL (R²/p)	***0.176***	***1.40E-06***	***0.146***	***2.25E-05***
**Current Cognitive Function**				
AttMech	*-0.161*	*0.064*	-0.013	0.952
CogFun	*-0.204*	*0.011*	-0.101	0.337
Age	*0.347*	*3.48E-06*	*0.357*	*4.56E-06*
AttMech: CogFun	-0.009	0.959	0.022	0.933
AttMech: Age	-0.012	0.952	0.060	0.646
CogFun: Age	-0.079	0.464	-0.026	0.870
AttMech: CogFun: Age	-0.105	0.338	-0.083	0.572
MODEL (R²/p)	***0.200***	***1.85E-07***	***0.138***	***4.82E-05***
**Trait Anxiety**				
AttMech	-0.048	0.731	0.033	0.831
TAnx	0.130	0.076	0.197	0.053
Age	*0.454*	*2.18E-08*	*0.462*	*3.24E-08*
AttMech: TAnx	0.092	0.439	-0.046	0.747
AttMech: Age	0.061	0.641	0.050	0.740
TAnx: Age	-0.113	0.232	-0.089	0.425
AttMech: TAnx: Age	0.136	0.172	0.076	0.556
MODEL (R²/p)	***0.205***	***1.26E-07***	***0.178***	***1.19E-06***
**State Anxiety**				
AttMech	-0.013	0.952	-0.016	0.952
SAnx	0.117	0.080	*0.146*	*0.044*
Age	*0.425*	*2.18E-08*	*0.400*	*3.27E-07*
AttMech: SAnx	*0.190*	*0.017*	*-0.082*	*0.569*
AttMech: Age	0.088	0.423	0.052	0.746
SAnx: Age	-0.092	0.319	-0.042	0.747
AttMech: SAnx: Age	*0.181*	*0.016*	0.011	0.952
MODEL (R²/p)	***0.214***	***5.97E-08***	***0.150***	***1.58E-05***
**Trait Depression**				
AttMech	-0.063	0.590	-0.035	0.814
TDep	0.135	0.064	0.123	0.227
Age	*0.464*	*2.18E-08*	*0.426*	*5.97E-08*
AttMech: TDep	*0.151*	*0.027*	-0.152	0.152
AttMech: Age	0.047	0.719	-0.010	0.956
TDep: Age	-0.096	0.342	*-0.175*	*0.044*
AttMech: TDep: Age	0.111	0.224	0.003	0.985
MODEL (R²/p)	***0.210***	***8.19E-08***	***0.193***	***3.27E-07***
**State Depression**				
AttMech	-0.093	0.348	-0.008	0.959
SDep	*0.187*	*0.038*	0.075	0.571
Age	*0.421*	*4.02E-08*	*0.395*	*3.47E-07*
AttMech: SDep	*0.169*	*0.040*	-0.051	0.740
AttMech: Age	0.034	0.800	0.070	0.615
SDep: Age	*-0.011*	*0.952*	-0.059	0.672
AttMech: SDep: Age	0.091	0.437	0.052	0.729
MODEL (R²/p)	***0.199***	***1.88E-07***	***0.135***	***5.97E-05***
**Usual sleep hours**				
AttMech	-0.116	0.229	-0.051	0.683
S/usual	-0.052	0.671	-0.082	0.427
Age	*0.415*	*4.02E-08*	*0.354*	*1.35E-06*
AttMech: S/usual	-0.083	0.423	0.021	0.899
AttMech: Age	0.013	0.952	0.058	0.644
S/usual: Age	0.057	0.629	-0.113	0.229
AttMech: S/usual: Age	-0.125	0.270	*-0.239*	*0.003*
MODEL (R²/p)	***0.175***	***1.64E-06***	***0.186***	***5.65E-07***
**Sleep hours the night before the session**				
AttMech	-0.114	0.229	-0.027	0.856
S/session	0.065	0.582	-0.104	0.272
Age	*0.428*	*2.35E-08*	*0.343*	*2.98E-06*
AttMech: S/session	*-0.160*	*0.044*	0.084	0.423
AttMech: Age	-0.018	0.929	0.044	0.740
S/session: Age	-0.055	0.641	*-0.146*	*0.026*
AttMech: S/session: Age	-0.077	0.563	*-0.156*	*0.018*
MODEL (R²/p)	***0.185***	***6.05E-07***	***0.189***	***4.54E-07***

SDep positively predicted the IEI during the attend faces condition (β = 0.16, *p* = 0.02; Supplementary Table 1; Supplementary Fig. 3E), showing that as current SDep increased, IEI increased. SDep did not interact with age (*p* = 0.95).

Finally, an interaction between age and S/session was observed for predicting IEI (β= -0.15, *p* = 0.026; Fig. 2C; Table 2; Supplementary Table 1), which explains a portion of the variance (R^2^ = 0.17, *p* = 1.34 × 10^− 7^, power = 0.67). Only for low and medium S/session groups, the simple linear regressions were significant (low: 4.9 ± 0.28, β = 0.63, *p* = 0.004; medium: 6.6 ± 0.45, β = 0.35, *p* = 4.79 × 10^− 5^; and high: 8.25 ± 0.53, β = 0.25; *p* = 0.09); as age increased, larger IEI.

### Which moderators affect the prediction of each attentional mechanism to WME (IEI)?

Table 2 shows the results of the moderation analysis including enhancement or suppression attentional mechanisms as the predictor variables, WME as the predicted one, and the following factors as moderators: years of schooling, current cognitive function, cognitive reserve, trait and state anxiety, trait and state depression, and usual hours and the night before the experimental session. The age factor was added as a covariate. Only the significant interaction between any of the attentional mechanisms and a moderator to predict IEI is reported.

#### The enhancement mechanism

**Fig. 3:**
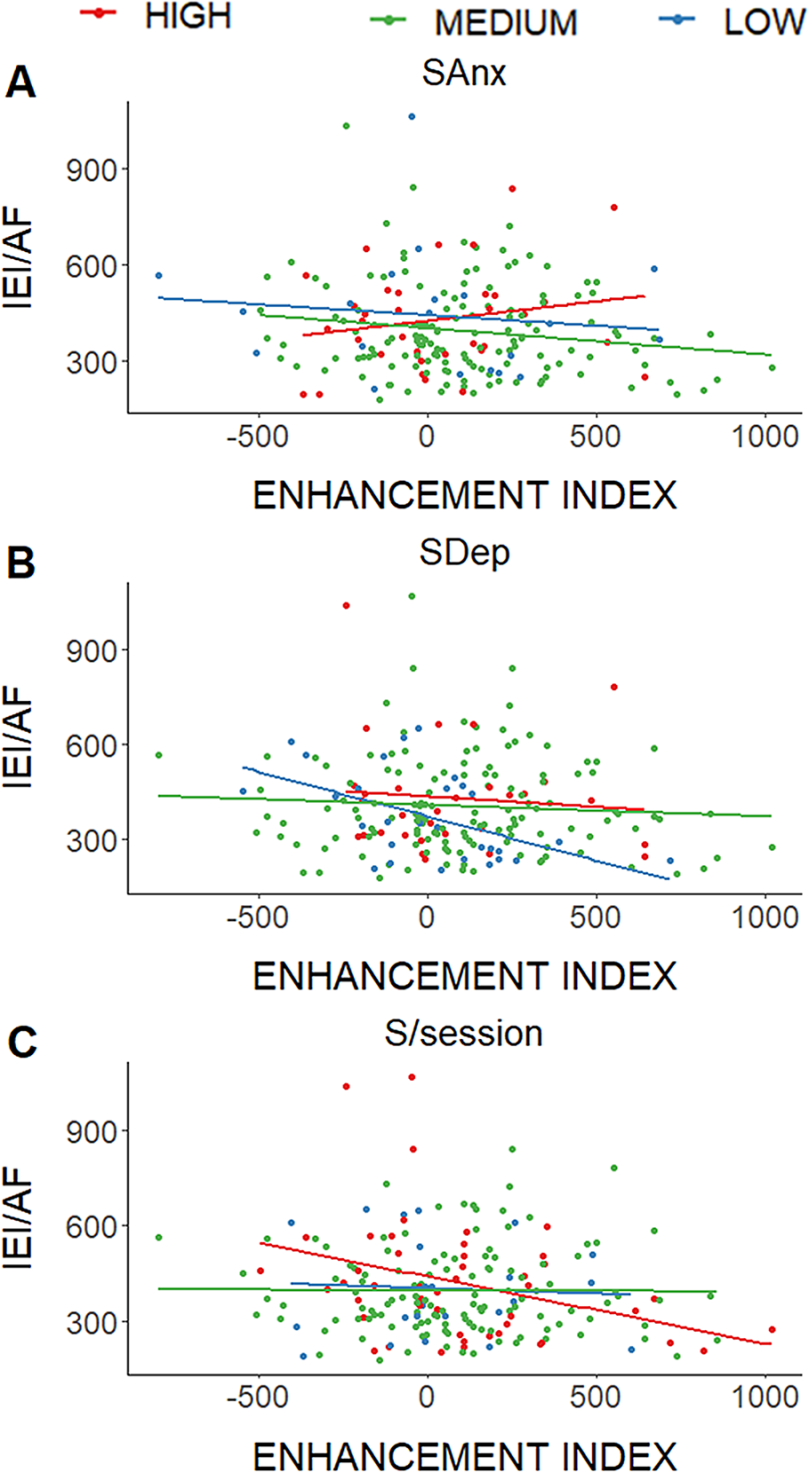
Moderation results of the significant interactions. (**A**) The enhancement index significantly predicted the IEI for attend faces condition, moderated by state anxiety (SAnx); only the high SAnx group showed that as the enhancement mechanism increased, the IEI significantly increased. (**B**) The enhancement index significantly predicted the IEI for the attend faces condition, moderated by state depression (SDep); only the low SDep group showed that as the enhancement mechanism increased, the IEI significantly decreased. (**C**) The enhancement index predicted the IEI for attend faces condition, moderated by sleep hours the night before the session; only the group that slept more hours the night before the session (high S/session; 8.25 ± 0.53 h) showed that as the enhancement mechanism increased, the IEI decreased. Graphs were performed with z-scores. For better understanding, X- and Y axes showed the raw data, corresponding ± 1SD of z-scores on X-axis, and ± 2SD of z-scores on Y-axis

The enhancement mechanism and SAnx interacted significantly to predict IEI (Table 2, β = 0.19, *p* = 0.017). This model explains a portion of the variance (R^2^ = 0.21, *p* = 5.97 × 10^− 8^, power = 0.90). Only for the high SAnx group (50.76 ± 5.76), as the enhancement mechanism increased, the IEI increased (β = 0.51, *p* = 0.028; Fig. 3A). In addition, an interaction among enhancement mechanism, age, and SAnx was observed (β = 0.181, *p* = 0.016). However, the sample size of the potential groups of this interaction was small enough to perform reliable analyses.

The enhancement mechanism and TDep significantly interacted (Table 2, β = 0.15, *p* = 0.027) for predicting IEI in the attend faces condition, which explains a portion of the variance (R^2^ = 0.21, *p* = 8.19 × 10^− 8^). However, no differences were found among slopes.

The enhancement mechanism and SDep significantly interacted (Table 2; Fig. 3B; β = 0.17, *p* = 0.04) for predicting IEI in the attend faces condition, which explains a portion of the variance (R^2^ = 0.2, *p* = 1.88 × 10^− 7^). Only for the low SDep group (31.14 ± 1.96), as the enhancement mechanism increased, the IEI significantly decreased (β= -0.56, *p* = 0.002; Fig. 3B).

Finally, the enhancement mechanism and S/session showed a significant interaction for predicting IEI in the attend faces condition (Table 2; Fig. 3C; β= -0.16, *p* = 0.044), which explains a portion of the variance (R^2^ = 0.18, *p* = 6.05 × 10^− 7^, power = 0.89). Only for the high S/session group (they slept 8.25 ± 0.53 h), as the enhancement mechanism increased, the IEI significantly decreased (β= -0.56, *p* = 0.0007).

#### The suppression mechanism

An interaction among the suppression mechanism, age and usual sleep hours (S/usual) was observed for significantly predicting IEI (β= -0.24, *p* = 0.003; Table 2), which explains a portion of the variance (R^2^ = 0.19, *p* = 5.65 × 10^− 7^, power = 0.89). However, the size of the possible groups of the interaction was not enough to perform reliable analyses.

Likewise, an interaction among suppression mechanism, age, and S/session was observed for predicting IEI (β= -0.156, *p* = 0.018; Table 2), which explains a portion of the variance (R^2^ = 0.19, *p* = 4.5 × 10^− 7^, power = 0.87). However, the size of the possible groups of the interaction was not enough to perform reliable analyses.

## Discussion

This study aimed to achieve three primary objectives. First, to determine whether age predicts the enhancement and suppression mechanisms of attention, and WME across adulthood. For the first time, our findings demonstrated that attentional mechanisms remain stable across adulthood. In contrast, WME declines with age, as previously reported [13, 14, 16, 23, 24, 26]. Furthermore, we examined the potential moderating effects of certain variables on the relationship between age and attentional mechanisms, as well as between age and WME. None of the moderators evaluated in this study influenced the relationship between age and enhancement mechanisms. However, CogRes moderated the relationship between age and suppression mechanisms (Fig. 2A).

CogRes is defined as the capacity of an individual to maintain or restore cognitive functioning in the face of aging or cerebral pathology [43]; it is developed throughout life via extended education, occupational engagement, cognitively stimulating, and leisure activities [44, 45]. The present findings indicated that only individuals with low CogRes exhibited a negative association between age and the suppression mechanism, suggesting that they are less effective in filtering out irrelevant information. Conversely, no age-related effect was observed on the suppression mechanism among individuals with medium or high CogRes. These findings align with prior evidence indicating a suppression deficit exclusively among individuals older than 60 [16]. Our results further elucidated that the suppression deficit intensifies with age, particularly affecting individuals with lower CogRes, whereas those with higher CogRes, even when over 60, remain unaffected.

In the present study, current CogFun served as a moderator and was evaluated using the MoCA, which assesses attention and concentration, executive functioning, memory, language, visuoconstructive skills, conceptual reasoning, calculation, and orientation. Current CogFun predicts WM performance—higher CogFun levels are associated with lower IEI, signifying enhanced WM capacity. It is plausible that, in accordance with overall cerebral function, current CogFun may mitigate memory deterioration, independent of chronological age.

Previous research has demonstrated that affective factors such as trait/state anxiety and depression are strongly correlated with variations in cognitive efficiency. For instance, individuals exhibiting elevated social anxiety levels have displayed increased N2pc amplitudes—a marker of selective attention within event-related potentials—when exposed to both angry and happy faces compared to neutral expressions [45–47]. In the current study, elevated trait and state anxiety and depression symptoms were associated with reduced WME (Supplementary Fig. 3), which concurs with existing literature [45–47]. However, when evaluating the specific impact of age on WME while controlling for anxiety and depression levels, it was revealed that individuals in the low and medium TDep groups demonstrated the expected age-related decline in WME (Fig. 2B). In contrast, the high TDep group exhibited diminished WME from an early age, indicating that the observed cognitive impairment in this group may be more attributable to elevated TDep levels rather than to aging itself.

Sleep has been acknowledged as a significant factor influencing cognitive performance [48–50], due to various physiological processes that occur during sleep, including synaptic homeostasis and restorative functions [49]. Sleep is essential for maintaining cognitive function and plays a role in delaying cognitive decline associated with aging [51]. In this study, participants were asked about the number of hours they slept the night prior to the experimental session (S/session) and their habitual sleep duration (S/usual). Only the S/session showed an interaction with age in predicting WME, specifically within the group reporting the shortest sleep duration, averaging 4.5 h (Fig. 2C). These results are consistent with previous findings demonstrating that short sleep duration negatively impacts WME [5, 51]. This suggests that sleep deprivation—defined here as sleeping approximately five hours—has a more substantial effect on older adults. Future research should examine the impact of fragmented sleep, insomnia, or other sleep-related disturbances that may impair WME [5, 51].

The second objective of this study was to determine whether attentional mechanisms could predict WME; however, our findings did not support this hypothesis. Numerous studies have reported that the presence of irrelevant information can deteriorate WME [13–16, 20, 23, 24, 26, 27].

Nevertheless, the final objective provided insight into the complex nature of the relationship between attentional mechanisms and WME, by considering the role of various moderating variables. In the case of the enhancement mechanism, SAnx, SDep, and S/session were found to moderate its relationship with WME. Specifically, among individuals in the highest SAnx group, an increase in the enhancement mechanism was associated with a decrease in WME (Fig. 3A). One possible explanation for this finding is that elevated anxiety may intensify superficial attentional engagement with facial stimuli, while simultaneously depleting the cognitive resources necessary for deeper encoding. This reduction in available cognitive resources may impair the specific recognition of facial stimuli, leading to diminished WME.

In contrast, for SDep, individuals in the lower depression group exhibited a positive association between the enhancement index and WME; as the enhancement index increased, WME also improved (Fig. 3B). This result may be explained by the idea that lower levels of depression are associated with greater cognitive resource availability, which in turn facilitates deeper processing and enhances both working memory and facial recognition abilities [47].

Regarding S/session, only individuals who reported an average of eight hours of sleep showed a positive relationship between the enhancement mechanism and WME. Specifically, increases in the enhancement mechanism were associated with improvements in WME (i.e., reduced IEI, Fig. 3C). This pattern was not observed in individuals who slept fewer than eight hours. These findings underscore the importance of sleep for cognitive function and suggest that when sleep duration approaches the recommended amount for adults [48], the enhancement mechanism and WME are more effective, which may facilitate performance in daily tasks.

Additionally, an interaction was observed among the suppression mechanism, sleep duration, and age; however, no conclusive analysis could be conducted due to the small sample sizes within the groups. Further research is warranted to confirm this interaction, and future studies should also consider including variables such as sleep quality, subjective restfulness, or objective assessments such as polysomnography.

This study presents several limitations. The research was conducted online during the COVID-19 pandemic, which restricted control over environmental variables due to variability in participants’ home settings. One of the inclusion criteria required participants to have a stable internet connection. However, factors such as lower income [52], advanced age [52, 53], and residence in rural areas [52] have been associated with limited broadband access. Consequently, this criterion may have excluded individuals with lower income and educational attainment. Nevertheless, in Mexico City—where this study was conducted—86% of households reportedly have Wi-Fi access [53]. Thus, there appears to be no clear association between internet connectivity and educational or socioeconomic status within this context.

Another limitation was the relatively low participation of individuals aged over 60 compared to younger participants. This disparity may be attributable to older adults’ perceived limitations regarding internet use, as supported by prior research [53].

Additionally, the study did not account for certain variables that may influence WME, such as socioeconomic status, occupational background, or retirement status [44, 54]. Future research may consider these factors as proxies for educational attainment and cognitive reserve, as they may impact WME and attentional mechanisms. Lifestyle-related factors, such as regular physical activity, should also be examined in greater detail. Physical activity has been shown to induce positive brain changes, including increased hippocampal volume [55], improved cognitive functioning [56], enhanced sleep quality and duration, and better mood and quality of life [57]. Subsequent studies may benefit from incorporating physical activity as a variable.

Furthermore, a notable limitation of the present study is the absence of data regarding the menstrual cycle phase and menopausal status in female participants. These factors may be relevant to cognitive efficiency, as evidence suggests associations between cognitive performance and menstrual cycle phases [58]. For instance, the luteal phase has been linked to poorer performance in emotion-related cognitive tasks [59]. Unfortunately, we were unable to assess estradiol/progesterone ratios. Future research should consider including these variables.

In conclusion, this study demonstrated that attentional enhancement and suppression mechanisms predict WME when age, lifestyle factors such as cognitive reserve and sleep duration, symptoms of anxiety and depression, and general cognitive function are taken into account. Accordingly, we propose that prioritizing adequate sleep, managing symptoms of anxiety and depression, and engaging in cognitively stimulating activities may support the preservation of attentional mechanisms and working memory efficiency in the aging process.

## Electronic supplementary material

Below is the link to the electronic supplementary material.

Supplementary Material 1

## Data Availability

No datasets were generated or analysed during the current study.
