# Supplementary material for: Predicting working memory efficiency across adulthood: the role of enhancement and suppression attentional mechanisms, moderated by age and other factors
Source: Aging Clin Exp Res. 2025 May 28;37(1):174. doi: 10.1007/s40520-025-03059-8 (PMC12119677; doi:10.1007/s40520-025-03059-8)
Supplement: Supplementary file 1 — Supplementary Material 1 [file 40520_2025_3059_MOESM1_ESM.docx]

**
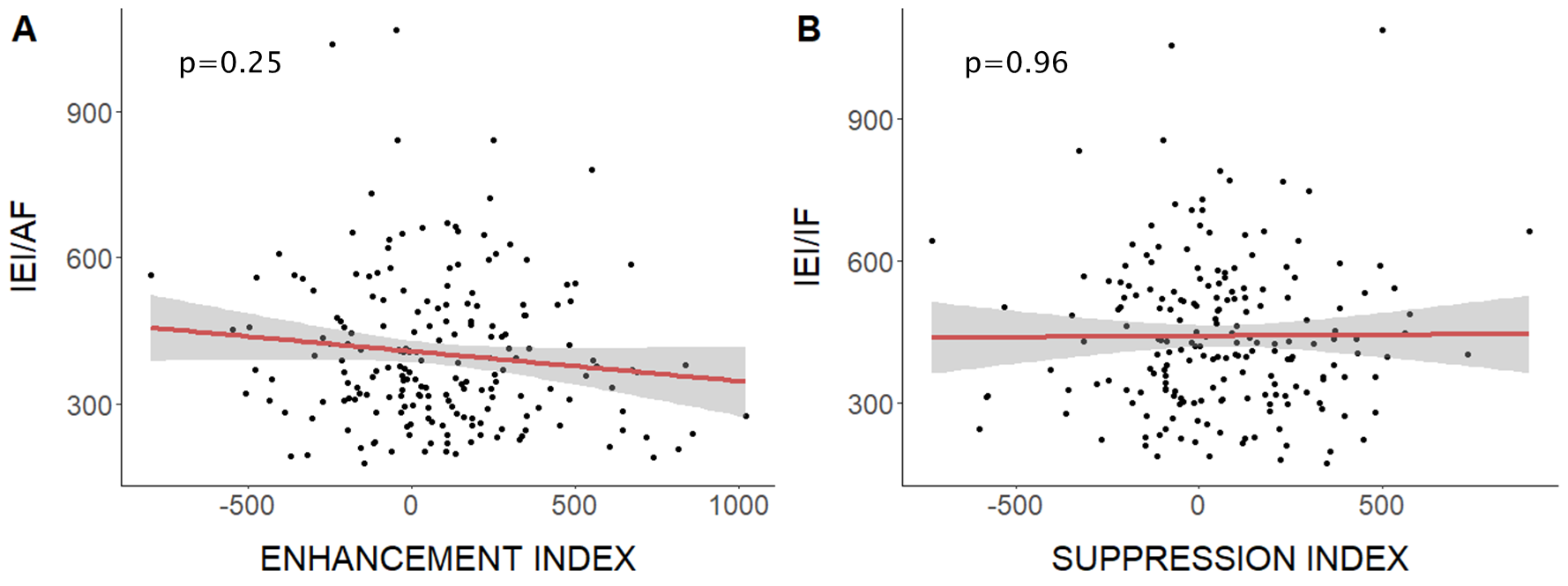
Supplementary Figure 1**. Plots of simple linear regressions for enhancement (A) and suppression (B) mechanisms as predictors of WME. Shaded areas correspond to the standard error.


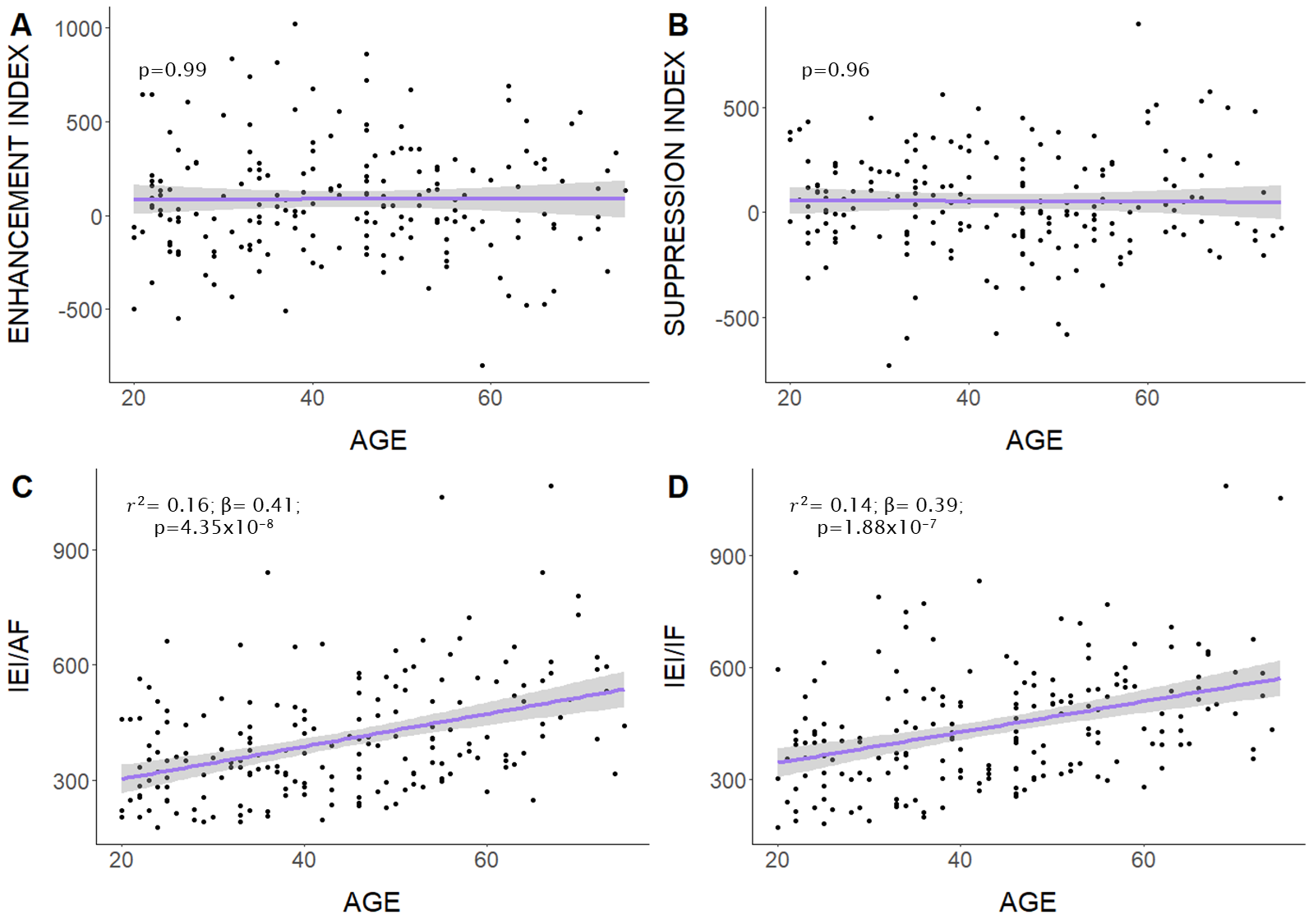
**Supplementary Figure 2**. Plots of simple linear regressions using age as the predictor variable for the enhancement index (A), the suppression index (B), and the WME (IEI) for the attend (C) and ignore (D) faces conditions. Age only significantly predicted WME, but not the attentional mechanisms. Shaded areas correspond to the standard error.

**Supplementary Table 1.** Attentional mechanisms and WME are predicted by age and the following moderators: years of schooling, current cognitive function, cognitive reserve, trait and state anxiety, trait and state depression, usual sleep hours, and sleep hours the night before the session. The R^2^ and p-value of the statistically significant models are indicated in bold, the significant effects of age and the statistically significant interactions are indicated in italics.

| **MODERATION** | **Enhancement - Age** | | **Suppression - Age** | | **IEI/AF - Age** | | **IEI/IF - Age** | |
| --- | --- | --- | --- | --- | --- | --- | --- | --- |
|  | **β** | **p** | **β** | **p** | **β** | **p** | **β** | **p** |
| **Years of schooling** | | | | | | | | |
| YS | 0.0799 | 0.5561 | 0.0250 | 0.8992 | -0.0811 | 0.4800 | -0.0451 | 0.7396 |
| Age | -0.0216 | 0.9190 | 0.0400 | 0.7783 | *0.4106* | *1.09E-07* | *0.3986* | *3.16E-07* |
| YS:Age | -0.0807 | 0.5902 | 0.2102 | 0.0435 | -0.0182 | 0.9456 | 0.0392 | 0.8005 |
| MODEL (R²/p) | -0.0081 | 0.8660 | 0.0257 | 0.1389 | ***0.1636*** | ***3.27E-07*** | ***0.1385*** | ***3.23E-06*** |
| **Cognitive Reserve** | | | | | | | | |
| CogRes | 0.1458 | 0.3793 | 0.0851 | 0.6554 | *-0.2346* | *0.0176* | -0.0057 | 0.9851 |
| Age | -0.0873 | 0.6294 | -0.0774 | 0.6595 | *0.5625* | *4.67E-08* | *0.3858* | *0.0002* |
| CogRes:Age | -0.1335 | 0.2517 | *0.2323* | *0.0140* | 0.0566 | 0.6720 | 0.0923 | 0.4383 |
| MODEL (R²/p) | 0.0006 | 0.6238 | ***0.0572*** | ***0.0102*** | ***0.1811*** | ***6.75E-08*** | **0.1446** | ***1.82E-06*** |
| **Current Cognitive Function** | | | | | | | | |
| CogFun | -0.0445 | 0.7469 | 0.0007 | 0.9927 | *-0.1887* | *0.0203* | -0.1086 | 0.2717 |
| Age | -0.0140 | 0.9522 | -0.0108 | 0.9522 | *0.3571* | *1.64E-06* | *0.3563* | *3.02E-06* |
| CogFun:Age | -0.0437 | 0.7469 | -0.0244 | 0.8992 | -0.0526 | 0.6595 | -0.0282 | 0.8535 |
| MODEL (R²/p) | -0.0122 | 0.9522 | -0.0151 | 0.9927 | ***0.1911*** | ***4.02E-08*** | ***0.1480*** | ***1.35E-06*** |
| **Trait Anxiety** | | | | | | | | |
| TAnx | 0.0139 | 0.9522 | -0.1068 | 0.3903 | 0.1203 | 0.2501 | 0.1754 | 0.0574 |
| Age | 0.0040 | 0.9851 | -0.0356 | 0.8254 | *0.4617* | *2.18E-08* | *0.4549* | *2.18E-08* |
| TAnx:Age | 0.0330 | 0.8404 | -0.1350 | 0.1937 | -0.1180 | 0.2140 | -0.0904 | 0.3793 |
| MODEL (R²/p) | -0.0147 | 0.9918 | 0.0049 | 0.5092 | ***0.1919*** | ***4.02E-08*** | ***0.1821*** | ***6.44E-08*** |
| **State Anxiety** | | | | | | | | |
| SAnx | -0.0622 | 0.6414 | -0.0805 | 0.5116 | *0.1324* | *0.0480* | *0.1390* | *0.0403* |
| Age | -0.0103 | 0.9522 | -0.0202 | 0.9238 | *0.4355* | *2.18E-08* | *0.4124* | *5.97E-08* |
| SAnx:Age | 0.0445 | 0.7396 | -0.1109 | 0.2574 | -0.0595 | 0.5902 | -0.0406 | 0.7396 |
| MODEL (R²/p) | -0.0093 | 0.8992 | 0.0028 | 0.5696 | ***0.1783*** | ***8.64E-08*** | ***0.1582*** | ***4.96E-07*** |
| **Trait Depression** | | | | | | | | |
| TDep | -0.0786 | 0.5707 | -0.0526 | 0.7307 | *0.1039* | *0.3254* | 0.1152 | 0.2572 |
| Age | -0.0235 | 0.9039 | -0.0162 | 0.9522 | *0.4542* | *2.18E-08* | *0.4379* | *3.24E-08* |
| TDep:Age | -0.0010 | 0.9927 | -0.1150 | 0.2869 | -0.1246 | 0.1808 | *-0.1716* | *0.0403* |
| MODEL (R²/p) | -0.0102 | 0.9272 | -0.0029 | 0.7190 | ***0.1883*** | ***4.35E-08*** | ***0.1892*** | ***4.35E-08*** |
| **State Depression** | | | | | | | | |
| SDep | 0.0296 | 0.8696 | -0.0901 | 0.4800 | *0.1610* | *0.0249* | 0.0508 | 0.7190 |
| Age | 0.0035 | 0.9851 | -0.0109 | 0.9522 | *0.4336* | *2.18E-08* | *0.3977* | *1.34E-07* |
| SDep:Age | 0.0315 | 0.8610 | -0.1481 | 0.1648 | -0.0087 | 0.9561 | -0.0532 | 0.6902 |
| MODEL (R²/p) | -0.0146 | 0.9915 | 0.0044 | 0.5219 | ***0.1827*** | ***6.30E-08*** | ***0.1439*** | ***1.93E-06*** |
| **Usual sleep hours** | | | | | | | | |
| S/usual | 0.0872 | 0.4460 | -0.1106 | 0.2869 | -0.0573 | 0.6294 | -0.0769 | 0.4766 |
| Age | 0.0032 | 0.9851 | -0.0129 | 0.9522 | *0.4059* | *5.97E-08* | *0.3880* | *1.82E-07* |
| S/usual:Age | 0.0431 | 0.7469 | -0.0047 | 0.9848 | 0.0622 | 0.5902 | -0.0922 | 0.3573 |
| MODEL (R²/p) | -0.0063 | 0.8005 | -0.0033 | 0.7293 | ***0.1630*** | ***3.27E-07*** | ***0.1509*** | ***1.05E-06*** |
| **Sleep hours the night before the session** | | | | | | | | |
| S/session | 0.1181 | 0.2572 | -0.1184 | 0.2572 | 0.0392 | 0.7469 | -0.1094 | 0.2516 |
| Age | 0.0168 | 0.9456 | -0.0238 | 0.8992 | *0.4158* | *4.35E-08* | *0.3727* | *3.76E-07* |
| S/session:Age | 0.0482 | 0.7293 | 0.0271 | 0.8696 | -0.0396 | 0.7469 | *-0.1662* | *0.0396* |
| MODEL (R²/p) | -0.0007 | 0.6527 | -0.0002 | 0.6414 | ***0.1592*** | ***4.55E-07*** | ***0.1733*** | ***1.34E-07*** |


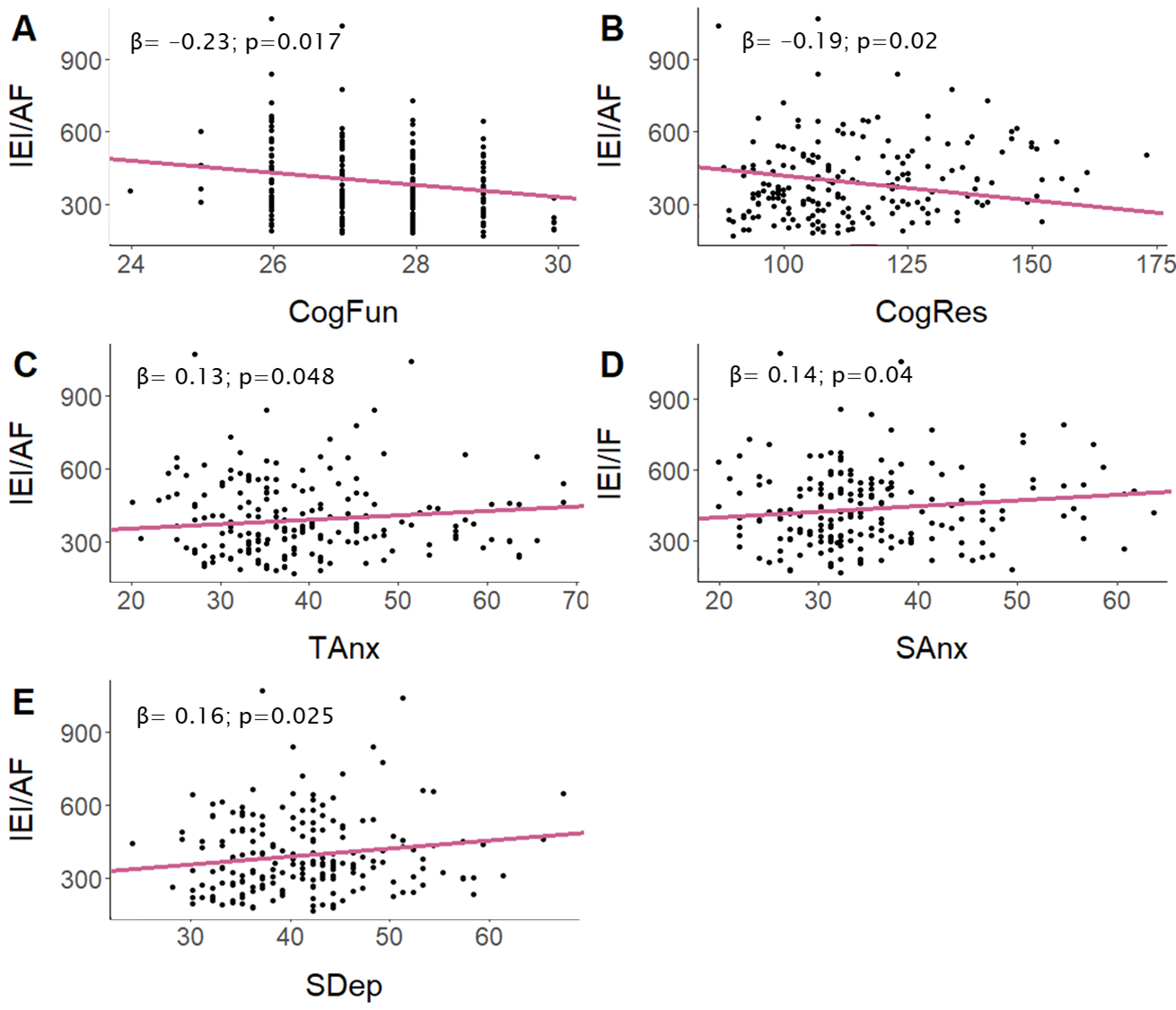


**Supplementary Figure 3.** Simple linear regression of the moderators predicting IEI. (A) Cognitive reserve during the attend faces condition. (B) Current cognitive function for the attend faces condition. (C) Trait anxiety for the attend faces condition. (D) State anxiety for the ignore faces condition. (E) State depression for the attend faces condition.
